# ^19^F NMR Functional Screening on a Benchtop NMR Instrument: Theoretical Analysis and Efficient Application to Drug Discovery

**DOI:** 10.1021/acs.analchem.5c07368

**Published:** 2026-05-05

**Authors:** Christina Jordan, Martial Piotto, Sandra Loss, Claudio Dalvit, Alvar D. Gossert

**Affiliations:** † Institute of Biochemistry, Department of Biology, 30845 ETH Zürich, Hönggerbergring 64, CH-8093 Zürich, Switzerland; ‡ Bruker Biospin, 34 Rue de l’Industrie, F-67166 Wissembourg, France; § Bruker Biospin, Industriestrasse 26, CH-8117 Fällanden, Switzerland; # Retired, private address: IT-38015 Lavis, Trento, Italy

## Abstract

Benchtop NMR instruments carry the potential to popularize and spread otherwise expensive NMR analyses, including applications in drug discovery. The main shortcoming of these affordable instruments is their limited sensitivity and resolution. Here, a functional assay based on ^19^F NMR on a commercial 80 MHz spectrometer is presented that reliably allows to determine the relative affinity of inhibitors over a large range of potency. An assay on the cancer-related enzyme thymidine phosphorylase was set up, and IC_50_ values for different inhibitors covering the range from nM to hundreds of μM were determined. The limitations and strengths of this n-FABS assay are evaluated theoretically in depth. Based on these insights, optimized assays with fluorinated substrates can be implemented, which provide an ideal opportunity for efficient and cost-effective functional assays on benchtop NMR instruments.

## Graphical abstract

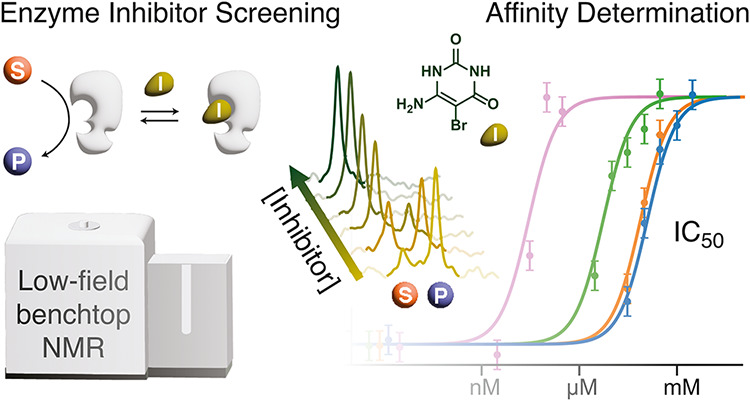

## Introduction

NMR functional assays for the identification of inhibitors of an enzymatic reaction and for the measurement of their IC_50_, i.e., the concentration of the inhibitor for which a 50% inhibition of the enzymatic reaction is achieved, have so far found limited applications. Other biophysical techniques are typically preferred due to their large throughput, which results in screening of many compounds in a relatively short measuring time. However, as previously discussed in detail,^1^ NMR functional assays, despite their lower throughput, have some important advantages which could make them an appealing alternative.

The ^19^F NMR functional assay, also known as n-FABS (n-Fluorine Atoms for Biochemical Screening), utilizes substrates or cofactors labeled with a fluorinated moiety.^1,2^ The n of the acronym stands for the number of magnetically equivalent fluorine atoms of the fluorinated moiety. ^19^F NMR spectra are recorded, and the ^19^F signals of the substrate and product of the enzymatic reaction are analyzed in the absence and presence of the tested potential inhibitors. The methodology has been applied with success to different enzymatic targets.^3–12^ Application of the n-FABS method on a benchtop NMR instrument would allow for a more widespread use of this methodology and, in particular, in laboratories with limited NMR equipment. The low sensitivity and small chemical shift dispersion at low magnetic fields represent major shortcomings for the efficient application of NMR as a functional assay. However, with an appropriate setup and a specific experimental design, these weaknesses can be overcome. To reiterate, two factors limit the application of NMR functional assays on a benchtop NMR instrument: (i) the limited separation in Hz between the signals of the substrate and the product of the enzymatic reaction and (ii) the low sensitivity of the instrument.

### Challenges of Benchtop Spectrometers I: Small Chemical Shift Separation

NMR functional assays performed with ^1^H detection can suffer from the limited dispersion in proton chemical shift, so that on a benchtop NMR instrument, the difference in Hz between the observed signal of the substrate and product could be very small. Furthermore, significant signal overlap originating from the buffer, substrate, cofactor(s), products, and tested molecule(s) is present at low magnetic field, so that often no proper quantitation of the product and substrate signals is possible. The use of n-FABS overcomes this limitation. The ^19^F chemical shift is very sensitive to the local environment, so that a large difference in ppm between the ^19^F NMR signals of the substrate and of the product containing the fluorine group is typically observed. This can occur even when the fluorinated moiety is located far from the site of the enzymatic modification. In cases in which the chemical shift difference is small, it is possible to increase it by adjusting some experimental conditions. These adjustments are feasible when n-FABS is performed in an end-point format. After quenching the enzymatic reaction, changes in the pH of the solution or the addition of cosolvents can be applied to increase the chemical shift separation.^1,13^

### Challenges of Benchtop Spectrometers II: Low Sensitivity

On standard high-field NMR equipment (500 to 600 MHz), direct binding experiments are the most prominent assay type. Here, ligand-based NMR screening is performed in the direct format, employing low concentrations of the tested fragments (30–200 μM) in order to maximize the fraction of bound fragment p_b_ defined as p_b_ = [EL]/[L_T_], with [EL] being the concentration of the protein-bound fragment and [L_T_] the total concentration of the tested fragment.^14,15^ This stringent experimental condition is not applicable to the low sensitivity of low-field instruments (typically 60 to 80 MHz), where a concentration of typically 1 mM of a small molecule is required for obtaining proton or fluorine spectra with sufficient signal-to-noise ratio for quantification within 15–30 min. Therefore, the ligand-based NMR screening in the direct or competition format cannot be performed in a reasonable measuring time on a low-field NMR instrument, unless the magnetization of the tested molecule or a reporter molecule is hyperpolarized. Several techniques for significantly increasing the sensitivity are available nowadays.^16–18^ Applications of these methods on a low-field NMR instrument have recently been reported in the literature.^19–21^ However, for this purpose, special equipment is required. Furthermore, direct detection of the ligand is limited to weak ligands, which exhibit fast binding exchange, such that inhibitors with *K*_D_ values below 1 μM can usually not be detected with these methods, unless one uses competition experiments.^22^

More flexibility is possible with functional NMR assays, in particular, with the n-FABS approach, where several experimental conditions can be optimized for improving the sensitivity and the detection limits, thus allowing efficient screening on a benchtop NMR instrument.

In this work, we present a theoretical analysis of n-FABS performed at low magnetic fields along with approaches to improve its sensitivity. The validity of the method is demonstrated experimentally using the enzyme thymidine phosphorylase (TP), where inhibitors with affinities ranging from nM to μM are characterized.

## Experimental Methods

### Sample Preparation

Enzymatic reactions were run in 500 μL of 200 mM KPH_4_O, pH 7.4, 0.5 units/ml of the enzyme thymidine phosphorylase, 10% D_2_O, and 0.11 mM DSS. The enzyme was preincubated with inhibitors at the concentrations stated in the manuscript for the individual experiments. The reaction was started by the addition of the substrate Trifluorothymidine (TFT) at a concentration of 1 mM. After 2 h at 37 °C, the reaction was quenched with 120 μL of 1 M HCl. The substrate TFT was purchased from Key Organics (Cat. No. HS-0007) as well as the inhibitors TP0001 (AS-56082), TP0003 (9X-0813), TP0004 (FS-3349), and TP0005 (SS-4601). TP0006 (224588–5G) and the TP enzyme (T2807) were purchased from Sigma-Aldrich, now Merck. See the Supporting Information for a detailed protocol of the reaction as well as structures and suppliers of the enzyme, inhibitors, and substrate. For the experiments with the paramagnetic agent, Gd­[DOTA]^−^ (DOTAREM 0.5 mmol/mL, Guerbet) was added after quenching the enzymatic reaction at 0.5 or 1 mM concentration, respectively.

### NMR Measurements and Data Processing

If not stated otherwise, NMR measurements of the quenched samples were performed using 5 mm NMR tubes in an 80 MHz spectrometer (Fourier80, Bruker) at 298 K. ^19^F spectra were measured with 256 scans with a time domain size of 3072 points, resulting in 1 s of acquisition time and 4 s of recovery delay (see Supporting Information for pulse sequence code). Processing was performed with a 2 Hz exponential window function. For spectra acquisition and processing, the software Topspin, versions 4.0.7, 4.5.0, and 5.0, was used.

### Data Analysis

Signal intensities were quantified by peak fitting with MestReNova v14.0.1 using the GSD method. After normalization of the integrals, the dose-response curves for each inhibitor were fitted with eq 6. For more details, see section “IC_50_ determination by dose-response” and Supporting Information.

## Results and Discussion

### Considerations for Setting Up an Enzymatic Assay by NMR

A molecule binding to an enzyme can inhibit the enzymatic reaction via a substrate-competitive, uncompetitive, or noncompetitive mechanism.^23^ The *F* value, which represents the percentage of inhibition for the three types of inhibitors, under the assumption of pure mechanisms, is given by

1 $$\begin{array}{l} c\mathrm{ompetitive}\;\mathrm{mechanism},F=\frac{100[I]}{K_{I,C}\left(1+\frac{\left[S\right]}{K_{M}}\right)+\left[I\right]} \end{array}$$

2 $$\begin{array}{l} u\mathrm{ncompetitive}\;\mathrm{mechanism},F=\frac{100\left[I\right]}{K_{I,U}\left(1+\frac{K_{M}}{\left[S\right]}\right)+\left[I\right]} \end{array}$$

3 $$\begin{array}{ll} n\mathrm{oncompetitive}\;\mathrm{mechanism} & F=\frac{100\left[I\right]}{K_{I,N}+\left[I\right]} \end{array}$$

where [S] and *K*_M_ are the concentration and the Henri–Michaelis–Menten constant of the substrate, respectively, and [I] and *K*_I_ are the concentration and the binding constant of the inhibitor, respectively. The *K*_M_ is given by

4 $$K_{M}=\frac{k_{\mathrm{off}}+k_{\mathrm{cat}}}{k_{\mathrm{on}}}$$

where *k*_off_ and *k*_on_ are the off-rate and on-rate constants, respectively, of the substrate binding to the enzyme and *k*_cat_ is the catalytic rate constant. See Figure 1 for a prototypical reaction scheme. Under rapid equilibrium conditions (i.e., *k*_off_ ≫ *k*_cat_), *K*_M_ corresponds in first approximation to the dissociation constant *K*_D_ of the substrate. This means that the lower the *K*_M_, the higher the enzyme’s affinity for the substrate. Substrates typically have *K*_M_ values in the μM to mM range. A balanced assay is referred to as an assay that ensures the efficient identification of all three different types of inhibition. This condition is achieved by using a concentration for the substrate which is comparable to its *K*_M_, i.e., [S]/*K*_M_ ≈ 1. If the *K*_M_ is in the few tens of μM range, then the balanced assay condition would require a substrate concentration of a few tens of μM, and this clearly is not feasible with a low-field NMR spectrometer without the use of hyperpolarization. Simulations of eqs 1 to 3 for different [S]/*K*_M_ ratios and at 50 μMconcentration for the inhibitor are shown in Figure 1 (for the simulation with the tested concentration of the inhibitor at 200 μM, which applies to fragment screening, see Supporting Figure 1).

**Figure 1:**
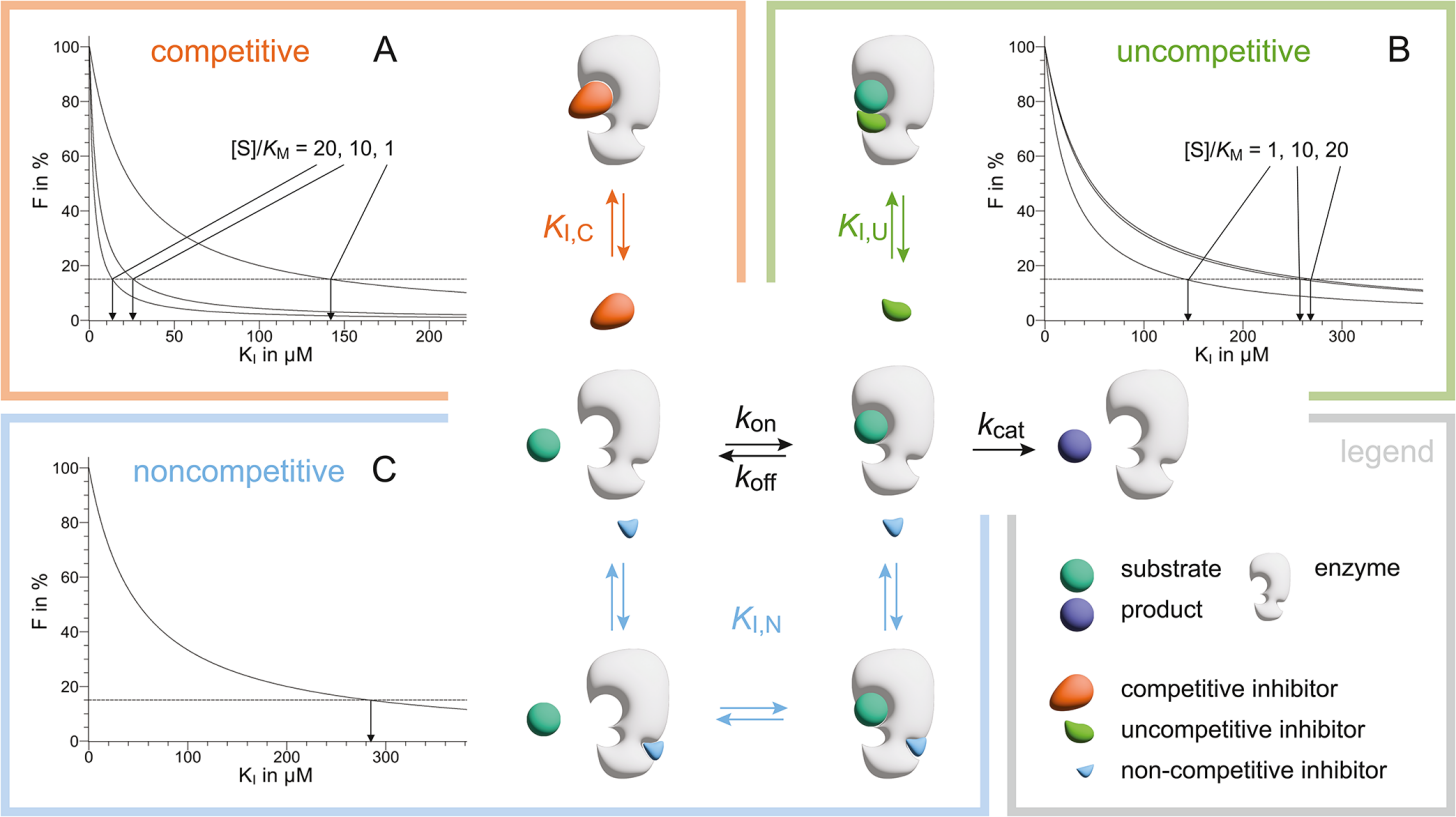
Scheme of an enzymatic reaction according to Henri-Michaelis-Menten kinetics, with three different competition mechanisms indicated. For each mechanism, a diagram is shown with the percentage of inhibition F as a function of the *K*_I_ of the tested inhibitor. The simulations were performed for (A) competitive mechanism with eq 1, (B) uncompetitive mechanism with eq 2, and (C) noncompetitive mechanism with eq 3. The experimental conditions with [S]/*K*_M_ = 1, i.e., balanced assay conditions, and [S]/*K*_M_ = 10 and 20, which represent assay conditions feasible on a benchtop NMR instrument when the *K*_M_ of the substrate is in the few tens of μM range, were simulated. The concentration of the tested inhibitors used in the simulation is 50 μM. The horizontal dashed line in the three graphs is drawn at *F* = 15% and represents the detection limit. Only molecules displaying an *F* value larger than 15% are considered hits. The detection limits for *K*_I_ for the three different inhibition mechanisms are shown in Table 1.

For example, if the *K*_M_ of the substrate is 50 μM, then for the balanced assay condition [S]/*K*_M_ ≈ 1, a substrate concentration [S] of 50 μM is required, a concentration that can be easily detected and accurately quantified on a high-field NMR instrument. In addition, the experimental conditions [S]/*K*_M_ = 10 and 20 were simulated, resulting in a substrate concentration [S] of 0.5 and 1 mM, respectively, which are well within the range observable on a low-field NMR instrument. The concentration of the tested inhibitors is set to 50 μM. F represents the percentage of inhibition, *K*_I_ is the inhibitory constant of the tested inhibitor, and the horizontal dashed line at *F* = 15% represents the limit of detection. The data are very reproducible when using n-FABS due to the lack of interferences from other substances and the fact that the simultaneous detection of both substrate and product signals allows the relative quantification of the two species, so that the inhibitory detection limit can be set to 15%. All of the molecules showing *F* > 15%, i.e., above the dashed horizontal line, are considered as hits.

The theoretical detection limits of the three different inhibitory mechanisms, substrate-competitive (A), uncompetitive (B), and noncompetitive (C), are described in Figure 1 and summarized in Table 1. In the presence of a competitive mechanism, the detection limit is for inhibitors with *K*_I,C_ < 142 μM 142 μMwhen the ratio [S]/*K*_M_ is 1, whereas this detection limit is reduced to inhibitors with *K*_I,C_ < 26 μM when [S]/*K*_M_ is 10 and *K*_I,C_ < 14 μM when [S]/*K*_M_ is 20. In other words, the inhibitor needs to compete with a higher concentration of substrate such that only relatively potent inhibitors are detected. The opposite trend is observed for the uncompetitive mechanism with detection of inhibitors with *K*_I,U_ < 142 μM when the ratio [S]/*K*_M_ is 1, *K*_I,U_ < 258 μM when [S]/*K*_M_ is 10, and *K*_I,U_ < 270 μM when [S]/*K*_M_ is 20. Here, substrate binding is a prerequisite for inhibitor binding, such that at higher substrate concentrations, more of the enzyme–substrate complex is present and relatively weak inhibitors can be detected. The detection limit for noncompetitive inhibitors does not depend on the ratio [S]/*K*_M_ but solely on the concentration of the tested inhibitor, as there is no competition or cooperative effect with the substrate. In the example of Figure 1C with the tested molecules at a concentration of 50 μM, the detection limit is reached for inhibitors with a *K*_I,N_ < 283 μM (the detection limits for all three inhibitory mechanisms using a concentration of 200 μM for the tested molecules are reported in the legend of Supporting Figure 1).

**Table 1:** Theoretical Detection Limits for n-FABS Assays for Different Inhibition Mechanisms and Different [S]/*K*_M_ Ratios^a^

	Mechanism	[S]/*K*_M_ = 1	[S]/*K*_M_ = 10	[S]/*K*_M_ = 20
	Competitive	*K*_I,C_ < 142 μM	*K*_I,C_ < 26 μM	*K*_I,C_ < 14 μM
	Uncompetitive	*K*_I,U_ < 142 μM	*K*_I,U_ < 258 μM	*K*_I,U_ < 270 μM
	Noncompetitive	*K*_I,N_ < 283 μM	*K*_I,N_ < 283 μM	*K*_I,N_ < 283 μM

a A detection threshold of *F* = 15% was assumed, and an inhibitor concentration of 50 μM was used (Figure 1). Due to the relatively low sensitivity of benchtop spectrometers, high substrate concentrations will mostly be required, typically leading to high [S]/*K*_M_ ratios.

It should be pointed out that the most frequent inhibitors are of the substrate-competitive type. The detection of this type of inhibitor, with n-FABS performed on a benchtop NMR instrument, can be improved, according to eq 1, by reducing the ratio [S]/*K*_M_. This can be achieved using two different strategies: (i) chemical approaches to either (i,a) improve signal intensity (reducing [S]) or (i,b) find substrates with increased *K*_M_. In addition, (ii) sensitivity improvements of the detection to reduce [S] can be sought.

#### Chemical Approaches for Increasing the Assay Window

Two different chemical approaches can be used for the purpose.

##### Use of Substrates Containing one CF_3_ Group or Multiple (2 to 4) Magnetically Equivalent CF_3_ Groups

The tagging of the substrate with these fluorinated moieties is nowadays chemically feasible (see, for example, refs 24–34 ). The fluorine NMR signal intensity of the substrate and of the fluorinated product of the enzymatic reaction originates from three (one CF_3_) to 12 fluorine atoms (4 magnetically equivalent CF_3_). This allows a reduction of the substrate concentration required for performing the n-FABS on a benchtop NMR instrument. In this case, the [S]/*K*_M_ ratio becomes smaller by reducing [S]. If the insertion of the multiple CF_3_ groups on the substrate results in a significant loss in solubility, the substrate’s solubility can be tuned by, e.g., chemically adding a functional group to the molecule at a position that is not in proximity of the site of the enzymatic reaction, as previously demonstrated.^25^ It should be pointed out that for some enzymes, the screening in the initial linear region of the enzymatic reaction requires the quenching when only a small fraction of substrate has been transformed into product. The use of magnetically equivalent multiple CF_3_ allows the detection of the small amount of generated product.^34^

##### Selection of Fluorinated Substrate with the Most Appropriate *K*_M_

In this approach, a few structurally closely related CF_3_-containing substrates are investigated. ^19^F NMR experiments in the presence of the enzyme and at different substrate concentrations are performed for measuring the *K*_M_ and *k*_cat_ of these CF_3_-containing substrates, as previously demonstrated.^25^ The substrate with a large *K*_M_ and sufficient enzymatic efficiency *k*_cat_/*K*_M_ is then selected for the n-FABS experiments. If the enzymatic efficiency of the selected substrate is very low, it is possible to overcome this limitation by increasing the concentration of the enzyme needed for the reaction and/or by increasing the incubation time of the enzymatic reaction before its quench. In this chemical approach, the reduction of the ratio [S]/*K*_M_ is achieved by increasing the *K*_M_ of the substrate.

#### Sensitivity Improvement of the NMR Measurement

##### Exploiting Stability of Quenched Reactions for Long Measurement Times

After quenching the enzymatic reaction, if the sample is stable, then, in principle, very long measurement times can be used to detect relatively low concentrations. This might open the opportunity for using the n-FABS for diagnostic purposes to monitor, for instance, the upregulation or downregulation of a specific enzyme in certain types of cancer. For the current assay, we opted for less than 30 min of measurement time in order to later be able to measure dose-response curves of inhibitors to determine affinities and thus use a substrate concentration of 1 mM. This results in measurement times of 2–3 h per dose-response curve or 5–8 inhibitor affinities that can be measured overnight.

The fact that reactions are quenched offers further advantages and flexibility: quenching performed on copies of the same sample at different incubation time points allows measuring the time course of the enzymatic reaction, or substances such as, for example, paramagnetic agents can be added to the quenched solution that could otherwise interfere with the reaction.

##### Use of Paramagnetic Relaxation Enhancement can Reduce Measurement Time 4-Fold

The use of paramagnetic relaxation enhancement (PRE) agents can decrease the T_1_ relaxation time substantially (following a suggestion by Reviewer 1).^35^ On high-field spectrometers, there is only a limited benefit due to the large ^19^F Chemical Shift Anisotropy (CSA) contribution to the T_1_ relaxation (according to experimental and calculated CSA values^36,37^ ). On low-magnetic field spectrometers, the CSA contribution to the T_1_ is significantly smaller, resulting in a longer T_1_. Therefore, it is anticipated that on a benchtop NMR instrument, the use of paramagnetic agents can decrease the measuring time. We tested the addition of 0.5 and 1 mM of Gd­[DOTA]^−^ to the samples relevant in this work (see Figure 3 for structures of the substrate and the product of the reaction). A concentration of 0.5 mM seemed to represent a suitable compromise between faster relaxation and limited line broadening. The CF_3_ group of the substrate and the product had respective T_1_ of 1.36 and 2.2 s in the absence of Gd­[DOTA]^−^ (Figure 2A). Addition of 0.5 mM Gd­[DOTA]^−^ shortened T_1_ to 0.31 and 0.4 s, respectively, such that the total repetition time could be reduced from 4 to 1.1 s, resulting in a reduction of the measurement time per sample from 20 to 5 min. For a sample containing substrate and product, spectra with essentially equal signal intensity were obtained in 5 min with 0.5 mM Gd­[DOTA]^−^ as in 20 min in the absence of the PRE-agent (Figure 2B). As it is important to be able to quantify the substrate and product in an enzymatic reaction for measuring IC_50_ values with precision, the resolution is important. The line width increased from 3.2 to 4 Hz for the substrate and from 2.5 to 3.3 Hz for the product, such that in the absence of PRE-agent, the signals are baseline separated, while in the presence of the PRE-agent, a slight overlap results. For the latter, proper integration of the product and substrate signals would require line shape fitting of the signals. Due to slightly higher expected precision in quantification in the absence of the PRE-agent, the data presented later in the manuscript were recorded without the PRE-agent. In general, the usage of the PRE-agent represents a viable approach that should always be tested for the system at hand. Even if this approach results in some overlap, it can be used for the initial screening phase when a large number of molecules have to be tested.

**Figure 2:**
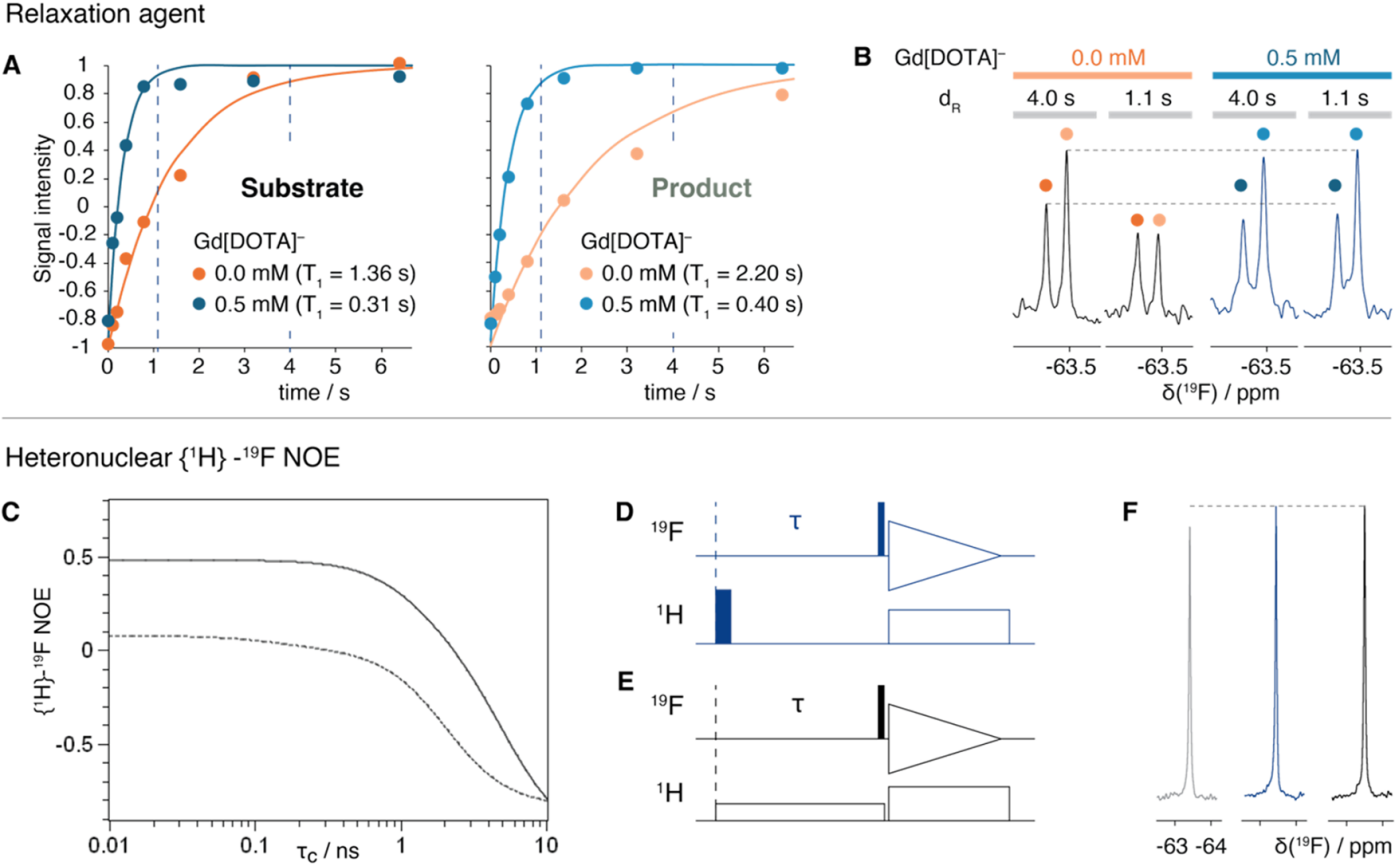
Signal enhancement by a PRE-agent and by heteronuclear ^1^H → ^19^F NOE. (A) ^19^F T_1_ relaxation curves of the CF_3_ group of the substrate and the product from inversion recovery experiments (see Figure 3A for the structures of the substrate and the product). Addition of 0.5 mM Gd­[DOTA]^−^ reduces T_1_ 4–5-fold. In (B), experiments performed on a sample containing substrate and product at a combined concentration of 1 mM are shown in the absence and presence of 0.5 mM Gd­[DOTA]^−^. The ^19^F downfield and upfield signals correspond to the CF_3_ resonance of the substrate and of the product, respectively. Experiments with total recovery delays (d_R_ = d_1_ + acquisition) of 4 and 1.1 s are shown for each condition. 256 scans were recorded, resulting in measurement times of 18 and 5 min for the long and short recovery delays, respectively. (C) Plot of the theoretically achievable heteronuclear {^1^H} → ^19^F NOE as a function of the correlation time τ_c_ derived from eq 6 in ref 38. The simulations were performed as described previously^38^ for a 600 MHz (dashed line) and an 80 MHz (continuous line) NMR spectrometer for an H–F spin system with r_FH_ = 2.6 Å and using the CSA values Δσ = 76.8 ppm and η_CSA_ = 0.^36,38^ The *x-*axis is on a logarithmic scale. The theoretical maximum for the NOE at 600 and 80 MHz fields is ∼0.07 and ∼0.48, respectively. (D, E) Pulse sequences used for performing the heteronuclear ^1^H → ^19^F NOE experiments via inversion of ^1^H followed by a delay τ prior to detection (D) or via saturation of ^1^H for a defined period τ prior to detection (E). Narrow and wide filled rectangles represent 90° and 180° pulses, respectively, and empty rectangles represent pulse trains for saturation and decoupling (Waltz16). (F) Actual NMR data obtained on a benchtop spectrometer for 10 mM of the substrate TFT in 90% H_2_O/10% D_2_O with a pulse-acquire experiment as reference (pulse sequence not shown, gray signal in F), pulse sequence D (blue), and pulse sequence E (black). The delay τ was set to 1 s in both experiments, and a recycle delay of 5 s was used. A total of 256 scans were recorded for each spectrum. Further experiments in D_2_O as a solvent can be found in Supporting Figure 2 as well as pulse sequence D, which was used throughout this work. All spectra were recorded on an 80 MHz NMR spectrometer (Bruker Fourier80) at 298 K.

##### ^1^H → ^19^F Heteronuclear NOE Enhances Signal by up to 10%

In this approach, an increase in the ^19^F NMR signal of a small molecule is obtained via ^1^H → ^19^F heteronuclear NOE. The heteronuclear NOE is achieved by applying a simple ^1^H hard 180° pulse followed by a delay (transient NOE) or by applying a ^1^H saturation scheme using a train of hard 120° pulses for a defined period (steady-state NOE). As it has been previously theoretically demonstrated with simulations, the signal enhancement for a small molecule is larger at a low magnetic field due to the reduced contribution of ^19^F CSA to the ^19^F longitudinal relaxation.^38^

It should be pointed out that the first ^1^H → ^19^F heteronuclear NOE experiments were performed by Solomon in the seminal paper published in the 50′s, where a tangible NOE was observed for the molecule HF.^39^ For an isolated H–F two-spin system, the maximum heteronuclear NOE achievable from proton to fluorine in the absence of ^19^F CSA contribution to the ^19^F longitudinal relaxation is 0.53 (i.e., 53% signal increase). Although in the paper the static magnetic field strength used for the experiments is not reported, it is evident that at that time the magnetic field was very low and, therefore, the ^19^F CSA contribution to the ^19^F R_1_ is extremely small. However, only a maximum heteronuclear NOE of ∼0.33 (∼33% signal increase) was observed experimentally, which is lower than the theoretical value of 53%.^39^

The ^19^F NMR spectra for a sample of Trifluorothymidine (TFT, see Figure 3 for the structure) in 90% H_2_O/10% D_2_O without and with {^1^H} → ^19^F NOE achieved with ^1^H inversion and ^1^H presaturation, obtained on an 80 MHz NMR instrument, are shown in Figure 2F. A signal increase of 8–10% is observed with the heteronuclear NOE step. This value is smaller than the predicted theoretical value for an isolated two-spin system. The major factor is surely that there is only one hydrogen in the vicinity of the CF_3_ group, at a relatively long distance of ∼3.2 Å, such that the weak enhancement from the ^1^H is divided among three ^19^F nuclei. A simple test on a molecule with four ^1^H nuclei in the vicinity of one ^19^F resulted in a higher enhancement of 14% (Supporting Figure 2). We further explored potential enhancements by measuring ^1^H → ^19^F NOE in D_2_O; however, the experiments resulted in a 5% lower enhancement. This indicates that H_2_O is an important contributor to the NOE-based signal enhancement, as it consistently adds ∼5% intensity to both tested molecules (Supporting Figure 2).

**Figure 3:**
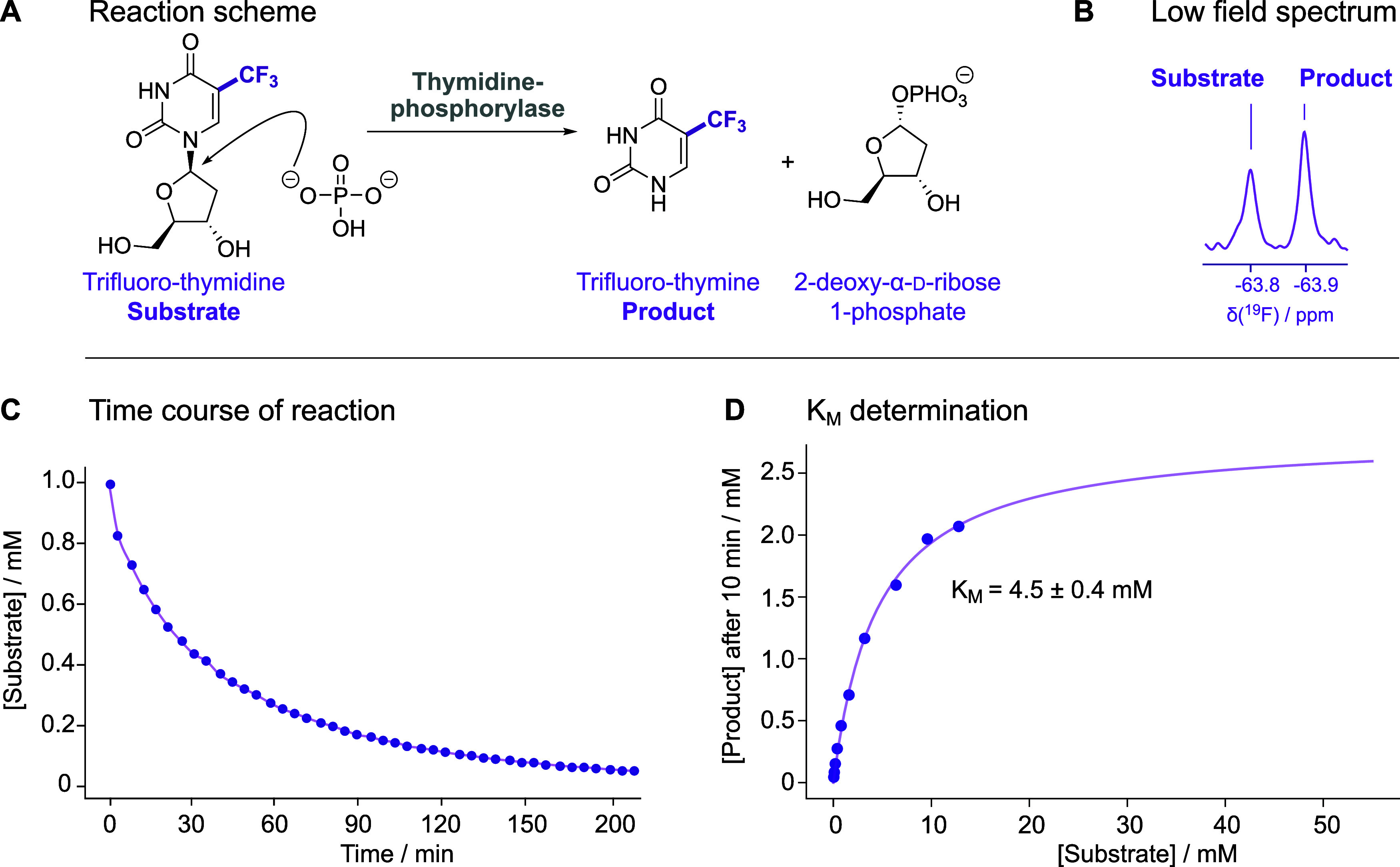
Reaction scheme and characterization of the enzymatic system. The thymidine-phosphorylase reaction is shown in (A), with *Substrate* and *Product* indicating the two substances observable in ^19^F NMR spectra. An example of a 1D ^19^F spectrum of the reaction without any inhibitor present, quenched after 1 h by the addition of HCl, and recorded on a benchtop 80 MHz NMR spectrometer (Bruker Fourier80) is shown in (B). The spectrum was recorded using 256 scans at 298 K with the pulse sequence shown in Figure 2D and a repetition time of 5.1 s. A time course of the reaction observed on the ^19^F signal of the substrate is shown in (C). In (D), the data used to determine the *K*_M_ value are plotted. The data for the plots shown in C and D were recorded on a high-field spectrometer (Bruker 600 MHz AVIIIHD spectrometer equipped with QCI-F cryoprobe). Reactions were carried out in aqueous solution at 298 K, in the presence of 200 mM NaPi in 90% H_2_O/10% D_2_O, at pH 7.2, using 0.5 units/ml of TP and 1 mM of the substrate TFT (for C). For D concentrations ranging from 50 μM to 12.8mM of TFT were used and each reaction was quenched after 10 minutes.

The theoretical enhancements are not reached, and this is most likely due to the presence of other relaxation mechanisms, the small but not negligible contribution of ^19^F CSA to the ^19^F longitudinal relaxation, and the internal rotation of the CF_3_ group, which will result in additional significant reductions of the enhancement even at low magnetic fields. Nevertheless, even a small signal enhancement of ∼10%, as observed in Figure 2F, is useful for reducing the measuring time of the experiments. For molecules smaller than 1 kDa with better-suited geometry, stronger enhancements can be expected, such that in general the pulse sequences in Figure 2D,E can be used for such experiments at low field.

### Characterization of the Enzyme System for Inhibitor Screening

The enzyme used in this study for the application of the n-FABS method on a benchtop NMR instrument is TP. TP is a member of the thymidine/pyrimidine-nucleoside phosphorylase family. The natural substrate of TP is thymidine, but it can also use trifluorothymidine (TFT, also called trifluridine) as a substrate, where it catalyzes the reversible phosphorolysis of TFT to trifluorothymine, i.e., the product containing the CF_3_ moiety, and 2-deoxy-D-ribose-1-phosphate (Figure 3A). TFT is used as a cancer drug as it incorporates into DNA and inhibits nucleotide synthesis. However, TFT is efficiently degraded by TP, which is often upregulated in cancer cells. The strong TP inhibitor Tipiracil® (later named TP0001) is used to block this resistance mechanism, and the parallel administration of TFT and Tipiracil® (Lonsurf®) is used for the treatment of patients suffering from metastatic gastric cancer, advanced gastric colorectal cancer, or gastroesophageal junction adenocarcinoma.^40–44^ In combination with bevacizumab (Avastin), it significantly prolongs the overall survival in patients with metastatic colorectal cancer.^45^

TFT has a CF_3_ group, and therefore, the ^19^F NMR signal intensity of the substrate (S) and of the fluorinated product 5-trifluorothymine (P) of the enzymatic reaction originates from three fluorine atoms.

Figure 3B shows the ^19^F NMR spectrum of the solution containing 1 mM TFT and 0.5 units/ml of *Escherichia coli* TP obtained on an 80 MHz NMR instrument using the pulse sequence from Figure 2D. The enzymatic reaction was quenched after 1 h with a concentrated solution of HCl. With these experimental conditions, the intensities for the substrate and product signals are similar. The signals of TFT and of the trifluorothymine product are clearly visible and well separated (Δδ = 0.166 ppm) even at this low magnetic field (Δν = 12.5 Hz), allowing rapid signal integration and making quantification of the individual species easily feasible. Both substrate and product are stable at low pH, thus allowing the acquisition of ^19^F NMR spectra even after several days from preparation. This would, in principle, allow very long measurements, but for practical reasons, we opted for 23 min of measurement time, which yielded a signal-to-noise ratio of 20 for the free substrate signal (as calculated by the sino routine in the software topspin, where the noise is defined as twice the standard deviation of a noise region in the spectrum).

To characterize the enzymatic reaction, a time course observation experiment was carried out to determine the enzymatic parameters and to find the optimal time for the best contrast between uninhibited and inhibited reactions, considering the limited signal-to-noise ratio of the benchtop instrument. Subsequent reactions were run with 0.5 units/ml TP for 2 h before quenching with HCl.

In order to determine the *K*_M_ of the enzyme, the product formation in the initial linear phase of the reaction was examined using a high-field NMR instrument. Initial product formation in the presence of different substrate concentrations revealed a relatively high *K*_M_ value of 4.5 ± 0.4 mM for TFT, which is 1 order of magnitude higher than the one for the natural substrate thymidine.^46^ Thus, the enzyme seems to have ∼10-fold lower affinity for TFT. Furthermore, there is a large difference in the *K*_M_ values of TFT for human TP and *E. coli* TP, as has also been observed for thymidine.^47^

This is a favorable system for performing the n-FABS on a benchtop NMR instrument since the *K*_M_ is very large. For the screening and IC_50_ measurements, a concentration of TFT of 1 mM is used, resulting in a ratio [S]/*K*_M_ = 0.22. These conditions allow efficient detection of all of the different types of inhibitors. Simulations using these experimental conditions for the substrate-competitive, uncompetitive, and noncompetitive inhibitors are shown in Figure 4 (left).

**Figure 4:**
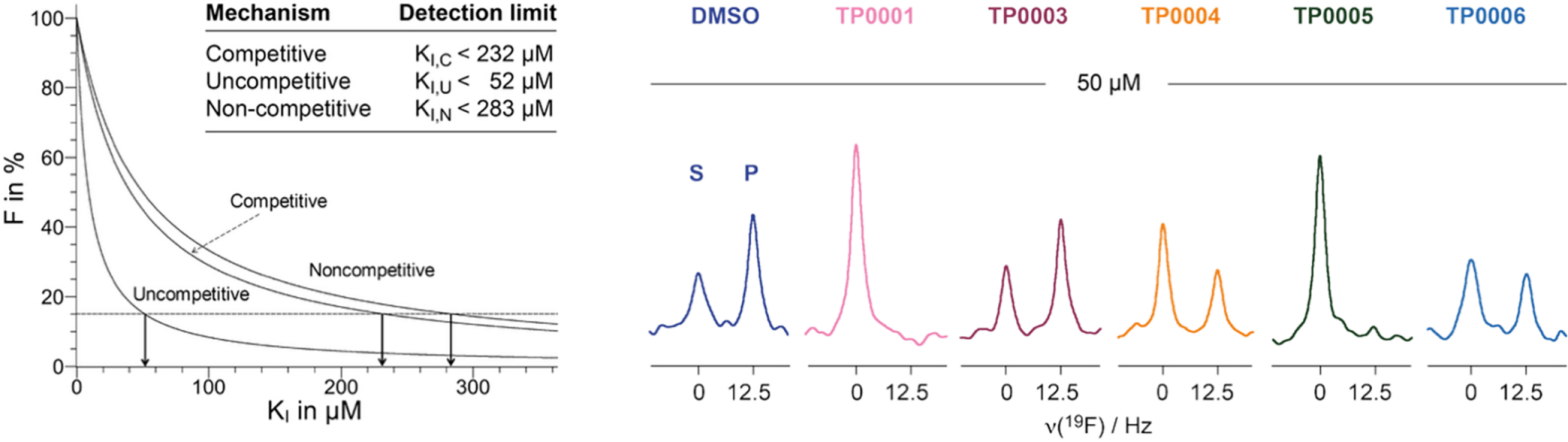
Single-point n-FABS screening experiments—theoretical detection limits and actual data on TP. Left: The percentage of inhibition *F* as a function of the *K*_I_ of the tested inhibitor is shown for the three mechanisms of inhibition with the assay conditions used in our study. The enzyme is *E. coli* TP, the substrate is TFT (1 mM) with a K_M_ of 4.5 mM, and the concentration of the tested molecules is 50 μM. The horizontal dashed line is drawn at *F* = 15% and represents the detection limit. Only molecules displaying an *F* value larger than 15% are considered hits. The detection limits for *K*_I_ listed in the table are indicated by arrows. Right: 1D ^19^F NMR spectra of the TFT-TP reaction at a single molecule concentration of 50 μM recorded on a benchtop spectrometer (Bruker Fourier80) are shown. Spectra were recorded using 256 scans at 298 K with the pulse sequence shown in Figure 2D and repetition time of 5.1 s (see the Supporting Information for details on sample preparation, inhibitor molecules, and pulse sequence). The ^19^F NMR signals of the substrate (S) and the product (P) are separated by 12.5 Hz (0.166 ppm), as indicated. These data were used to triage the compounds and to determine the titration range in subsequent dose-response experiments.

### Screening and Single-Point Ranking of Identified Inhibitors

In the first step, a number of candidate inhibitors were tested in a single-point assay. All molecules were added at a concentration of 50 μM to the reaction (200 mM KPH_4_O, pH 7.4, 1 mM TFT, 0.5 units/ml thymidine phosphorylase, 50 μM tested molecule, 10% D_2_O, 0.11 mM DSS; for further details, see the Supporting Information). After 2 h at 37 °C, the reaction was quenched with HCl, and ^19^F spectra were measured (Figure 4). These single-point measurements required 256 scans, i.e., ∼25 min per compound, and were used to triage them for subsequent dose-response experiments (It should be pointed out that the same experiments performed on a 600 MHz instrument equipped with a cryogenically cooled ^19^F probe would require just 1 scan.). Compounds TP0001 and TP0005 showed complete inhibition of the reaction, while compound TP0003 did not show any significant inhibition. TP0003 was dropped at this point because it did not show sufficient inhibition.

### IC_50_ Determination by Dose-Response

In a subsequent step, compounds TP0001 (Tipiracil), TP0004, TP0005, and TP0006 were measured in a dose-response format. To this end, the IC_50_ was estimated for each compound from the single-dose experiment. The percentage of inhibition (*F*) can be calculated from the integrals of the ^19^F NMR signals of the product (*P*) or substrate (*S*), as previously reported, and from this extracted value, in the absence of allosteric effects, an approximated estimation of the IC_50_, i.e., the concentration of the tested molecule required for achieving 50% inhibition of the enzymatic reaction, can be derived, according to the following equation

5 $${\mathrm{IC}}_{50}=\left(\frac{100-F}{F}\right)\left[I\right]$$

where [I] is the concentration of the tested molecule. This is feasible when *F* is smaller than 100%. For molecules TP0001 and TP0005, total inhibition is observed at a 50 μM concentration, and therefore, the IC_50_ value cannot be extracted from the single-point measurement. For molecules TP0004 and TP0006, partial inhibition is observed at 50 μM concentration (Figure 4), and their IC_50_ values calculated with eq 5 are 84 and 121 μM, respectively.

For a more precise measurement of the IC_50_ value of the weak inhibitors TP0004 and TP0006 and for measuring the IC_50_ of the two potent inhibitors TP0001 and TP0005, dose-response experiments at five different concentrations of the identified hit were performed. The F values extracted from these spectra according to the procedure described in ref 1 were then plotted as a function of [I], and the data were fitted with the following equation

$$F=P\cdot \left(1-\frac{1}{1+{\left(\frac{\left[I\right]}{{IC}_{50}}\right)}^{n}}\right)$$ 6

where *n* is the cooperativity factor (known also as Hill slope), which in the absence of cooperativity is 1, and *P* is the maximum response, here 100%. During the fitting procedure, *n* and *P* are fixed or allowed to float in order to see if the fitted values are close to 1 and 100, respectively.

Alternatively, one can quantify the integral of the substrate signal and use eq 7 for extracting the IC_50_ value.

7 $${\left[S_{+}\right]}_{t}=\frac{{\left[S_{-}\right]}_{t}-{\left[S\right]}_{0}}{1+{\left(\frac{[I]}{{\mathrm{IC}}_{50}}\right)}^{n}}+{\left[S\right]}_{0}$$

where [S_+_]_t_ is the concentration of the substrate in the presence of the tested molecule, [S_–_]_t_ is the concentration of the substrate in the control experiment, i.e., without the tested molecule, and [S]_0_ is the concentration of the substrate used for the experiments. During the fitting procedure, *n* and [S]_0_ are fixed or allowed to float in order to see if the fitted values are close to 1 and the total substrate concentration, respectively.

The IC_50_ values calculated using eq 6 and keeping *n* = 1 and *P* = 100 are shown in Figure 5. Within the experimental error, the same IC_50_ values were obtained, regardless of whether eqs 6 or 7 were used. Also, including *n* and *P* as fitted variables in eq 6 led to the same results within the stated error. In the Supporting table, the results from all fitting scenarios are shown.

**Figure 5:**
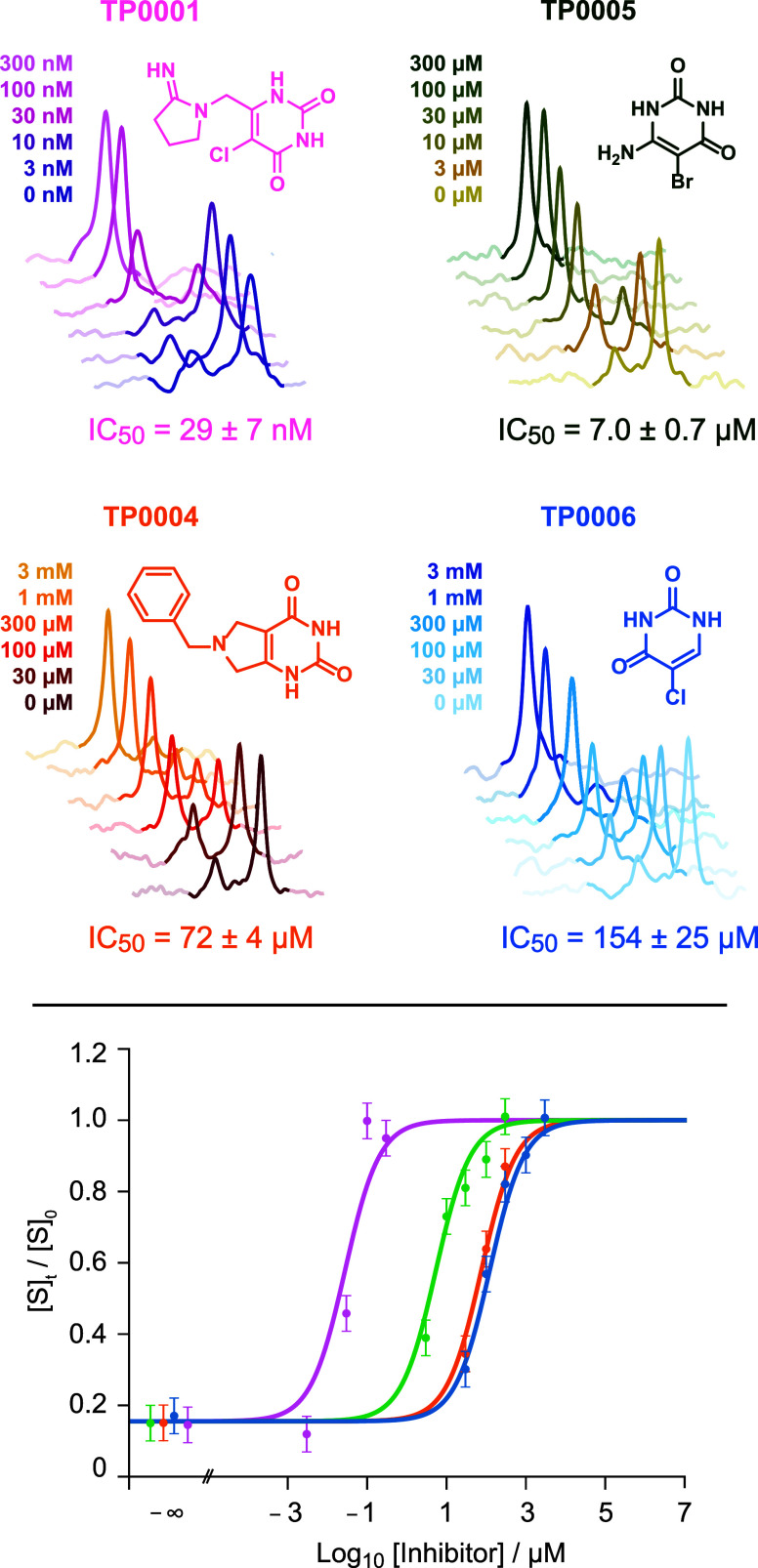
Dose-response curves for the inhibitors identified for TP. A series of 1D ^19^F NMR spectra obtained on a benchtop 80 MHz NMR spectrometer (Bruker Fourier80) at different inhibitor concentrations are shown for each inhibitor. Note the adapted concentration ranges for each inhibitor based on the single-point data from Figure 4. The compound structure and its number are given next to the spectra. The bottom graph shows the logarithmic dose-response curves of the four tested inhibitors in the same color code as above. [S]_0_ is the substrate concentration before the reaction, [S]­t is the concentration after 2 h incubation time in the presence of different amounts of inhibitor. The error bars reflect the spectral noise, as determined by the *sino* routine of the software TopSpin. IC_50_ values shown for each compound were extracted from the fitted curves using eq 6 (See Supporting Figure 3 and Table Table 1 for further details).

The IC_50_ values that were determined for the different inhibitors span several orders of magnitude, from 29 nM to >100 μM, demonstrating the wide detection range of the n-FABS assay. The compounds are all analogues of thymine, carrying different modifications such as halogenation or the addition of a positively charged amine. Both of these modifications are known to increase the affinity for thymine. While halogenation has a small effect, the addition of a positively charged group enhances binding significantly, as it mimics the transition state, which carries a partial positive charge on the C1 of the ribose. The trend in binding agrees well with published IC_50_ values for similar compounds in the literature.^48^ For TP0001 and TP0005, IC_50_ values were determined for human TP, with 35 nM and 17 μM, respectively,^48^ which agrees well on a relative and an absolute scale. The value for TP0001 probably represents the lower detection limit of this particular assay, as it is close to the enzyme concentration employed here.

### Comparison of IC_50_ Values with Other Types of Assays

The IC_50_ values extracted with eq 6 depend on many factors and are, by definition, not comparable between different assays. In this assay, which is optimized for benchtop NMR, several factors can lead to a larger IC_50_ value (i.e., weaker inhibition value) than what would typically be obtained with a conventional biochemical assay. For comparison: enzymatic assays, with highly sensitive fluorescent readout formats, for instance, are typically run with much lower substrate concentrations (in the μM range) and only during the initial phase of a reaction (5–10% turnover), where simple Michaelis–Menten kinetics apply. However, on a benchtop NMR instrument, the sensitivity is too low to reliably quantify differences below 1%, even with high substrate concentrations, such that the reaction must be run to a much later time point with high turnover. Based on this comparison, the consequences of the difference in substrate concentration and turnover deserve a discussion.

The first difference is the high concentration of the substrate that is used. In general, higher inhibitor concentrations in the competitive case displace higher substrate amounts. This is particularly relevant when the *K*_M_ of the substrate is small. However, in the current case, this does not represent a limitation of the assay, as the substrate has very weak affinity to the enzyme. The resulting IC_50_ values in this NMR assay format for substrate-competitive inhibitors are slightly higher than those extracted from an enzymatic assay performed with fluorescent readout (approximately 20%). For lower *K*_M_ values, however, while the relative ranking is still preserved, the IC_50_ values can change by an order of magnitude when changing the substrate concentration from 10 to 1000 μM. It should be pointed out that the opposite trend would be observed for the IC_50_ values of substrate-uncompetitive inhibitors.

The second difference stems from running the enzymatic reaction beyond the initial phase, i.e., where the velocity is constant and there is a linear relation between the free enzyme concentration and the product formed.^49^ In the present case, the reaction proceeds well beyond the linear phase (Figure 3), i.e., the reaction is run until 80% substrate conversion. In this regime, as an example, reducing the enzyme concentration by half does not lead to 50% less substrate turnover; only 30% less substrate is turned over. This is due to the nonlinear behavior of the enzymatic reaction and leads to a 2.2-fold higher IC_50_ value compared to a biochemical assay with only 5% substrate turnover.^49^ The IC_50_ values determined in this work, however, agree well with the literature because there the reaction seems to have been run longer in order to discern the small difference in the UV spectrum.

In comparison with other NMR-based assays, n-FABS has a few highly favorable properties. Direct *K*_D_ determination by standard NMR on expensive high-field instruments requires mg quantities of isotope-labeled protein and similar measurement times. Notably, *K*_D_ determination is limited to affinities weaker than 1 μM, while in n-FABS, the limit for *K*_I_ of the inhibitor is determined by the enzyme concentration, which is here in the low nM range, such that in the n-FABS approach, inhibitors with *K*_I_ values from single-digit nM to mM can be characterized using much lower quantities of far less expensive reagents. Clearly, for measuring *K*_I_ values in the millimolar range, compounds need to be soluble at this concentration in aqueous solution. If n-FABS is performed on cutting-edge NMR equipment, for example, on a 600 MHz instrument equipped with a cryogenically cooled ^19^F probe, a measurement requires just a few seconds for the screening of each molecule and a few minutes for determining an IC_50_ value. Furthermore, the substrate concentration used in the assay performed on high-field NMR instruments is significantly lower, thus typically not requiring chemical optimization of the substrate.^50^ This must be balanced against the much more affordable price of a benchtop instrument.

## Conclusion

In conclusion, we present the application of the n-FABS method on a benchtop NMR instrument for identifying weak and strong inhibitors against an enzyme and measuring their IC_50_ values. In the case presented, hardly any assay development was required. However, for less favorable enzyme systems, we laid out the theoretical foundation for different optimization strategies. The assay is not high throughput, as it requires about 15–30 min of measurement time for the screening of each tested molecule and three hours for determining an IC_50_ value. As shown experimentally, the measuring time can be reduced by a factor of ∼4 if a paramagnetic agent is used, resulting in an increased throughput. Therefore, IC_50_ values for a set of 5–10 compounds can comfortably be measured overnight, a number of compounds that often matches the chemistry output in hit-to-lead optimization rounds. In this assay, affinities spanning the range from mM to low nM can be determined, which corresponds to the relevant range for drug development. Thus, this inexpensive approach is well-suited for enabling drug discovery efforts in laboratories with limited NMR resources.

## Supplementary Material



## Notes

Additional experimental details and protocols, detection limits for inhibitors tested at higher concentrations, alternative fitting results, and pulse sequence (PDF)
