# Supplementary material for: 19F NMR Functional Screening on a Benchtop NMR Instrument: Theoretical Analysis and Efficient Application to Drug Discovery
Source: Anal Chem. 2026 May 5;98(19):14049–59. doi: 10.1021/acs.analchem.5c07368 (PMC13191725; doi:10.1021/acs.analchem.5c07368)
Supplement: Supplementary file 1 [file ac5c07368_si_001.pdf]

Supplementary Material to

^{19}F NMR Functional Screening on a Benchtop NMR Instrument: Theoretical Analysis and Efficient Application to Drug Discovery

Christina Jordan,[a] Martial Piotto,[b] Sandra Loss,[c] Claudio Dalvit*[,d] & Alvar D. Gossert*[,a]

[a] Institute of Biochemistry, Department of Biology, ETH Zürich, Hönggerbergring 64, CH-8093 Zürich, Switzerland
[b] Bruker Biospin, 34 Rue de l'Industrie, F-67166 Wissembourg, France
[c] Bruker Biospin, Industriestrasse 26, CH-8117 Fällanden, Switzerland
[d] IT-38015 Lavis, Trento, Italy
* Corresponding authors: cdalvit9@gmail.com, alvar.gossert@biol.ethz.ch

Table of contents:

Methods: Protocol for preparation of thymidine phosphorylase reaction. 2
Detection limits for inhibitors tested at 200 μM concentration 4
Signal enhancement by heteronuclear $^1H \rightarrow {}^{19}F$ NOE... 5
Example of IC_{50} fitting procedure with equations 8 and 9 on TP0006..................................... 7
IC_{50} values determined with different fitting parameters 8
Pulse sequence 9

Methods: Protocol for preparation of thymidine phosphorylase reaction.

In general, for most reproducible results it is important to use one “Master Solution”, which contains all substances common to each experiment (ddH_2O, buffer, enzyme solution and D_2O+DSS). This reduces variations from e.g. pipetting of mixing (Figure 1).

Always include at least one reaction without inhibitor as control.

Protocol for 500 µl reactions (can be scaled to lower or higher volumes).

1. Prepare a sufficient amount of master solution (see recipe below)
2. Pipette 450 µl of the stock into each separate tube
3. Add *n* µl of the inhibitor (i.e. 0.5 µl of 50 mM stock for 50 µM)
4. Add 10 µl of TFT stock solution into each tube
   The reaction starts now – move fast to points 5 and 6.
5. Mix well by pipetting up and down or using a vortex mixer
6. Incubate the samples at 37 °C for 2 h
   If possible use a shaking block at 1000 rpm
7. Quench each reaction by adding 120 µl HCl (1 M) and mix well.
   Samples are long-term stable at 4°C
8. Pipette 550 µl of each sample into a separate 5 mm-NMR-tube for measurement

Concentrations in reaction

200	mM	KPH_4O, pH 7.4
1	mM	TFT
0.5	units	Thymidine phosphorylase
0–3000	µM	Inhibitor
10	%	D_2O
0.11	mM	DSS

Recipe for one sample
(first 4 items are pre-prepared as master solution)

200	µl	Buffer (0.5 M KPH_4O, pH 7.4)
50	µl	D_2O + DSS (10% of the total volume)
100	µl	Enzyme solution (2.5 units/ml)
140–*n*	µl	ddH_2O
n	µl	Inhibitor stock solution (50 mM in dDMSO)
10	µl	TFT (50 mM in dDMSO)

Compound code	Vendor	Cat. number	Structure
TP0001	Key Organics	AS-56082	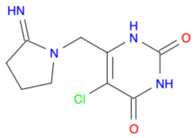
TP0003	Key Organics	9X-0813	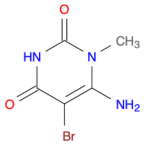
TP0004	Key Organics	FS-3349	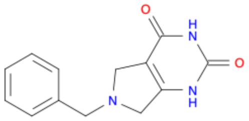
TP0005	Key Organics	SS-4601	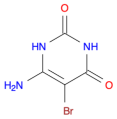
TP0006	Sigma-Aldrich	224588-5G	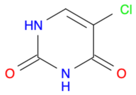
Trifluridine (TFT)	Key Organics	HS-0007	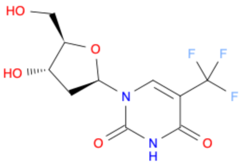
Thymidine phosphorylase *(E. coli)*	Sigma-Aldrich	T2807	

Detection limits for inhibitors tested at 200 μM concentration

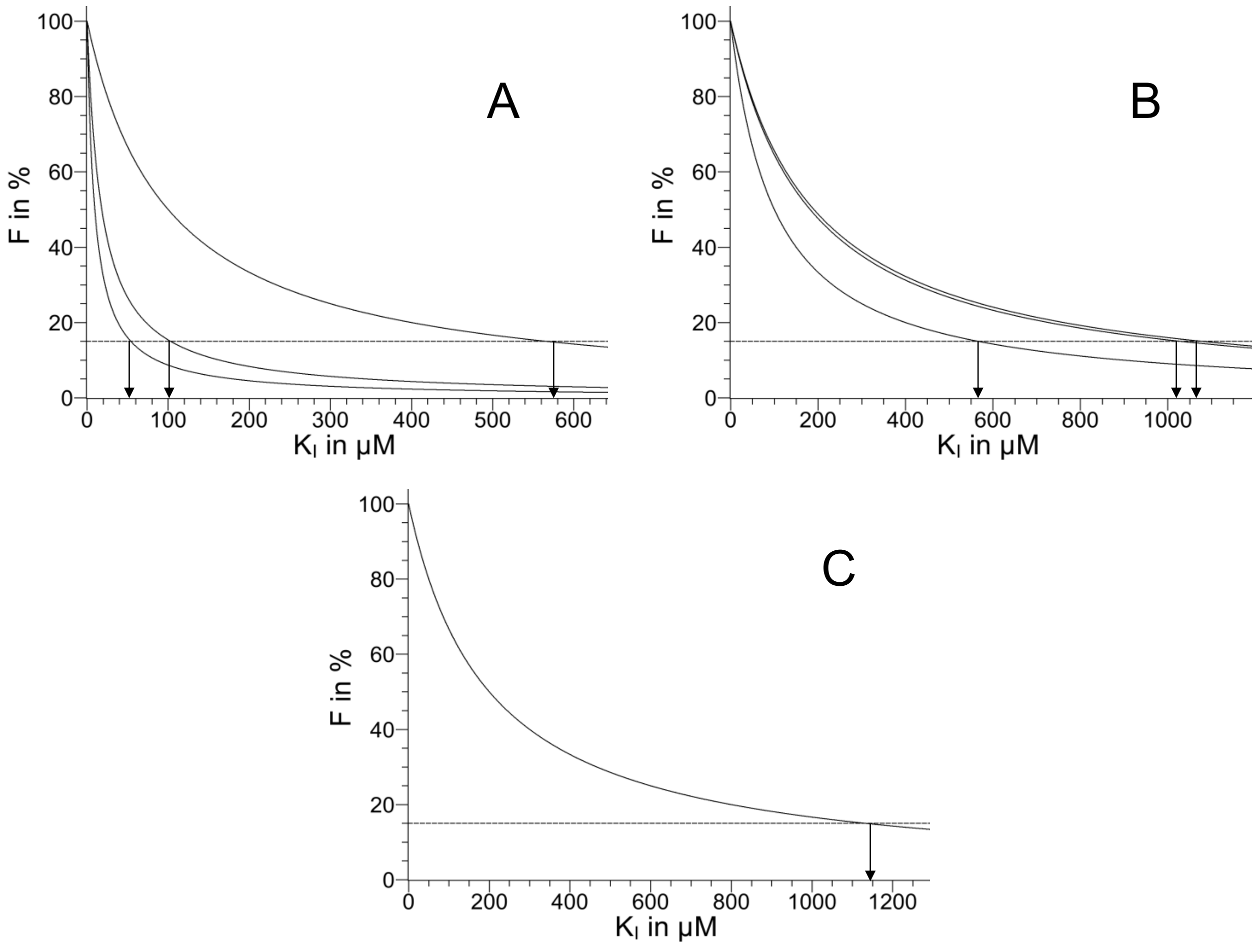

Suppl. Figure 1: Percentage of inhibition F as a function of the K_I of the tested inhibitor and for the three mechanisms of inhibition. The simulations were performed for (A) competitive mechanism with equation (1), (B) uncompetitive mechanism with equation (2) and (C) noncompetitive mechanism with equation (3). The experimental conditions with $[S]/K_M = 1$ i.e. balanced assay condition and $[S]/K_M = 10$ and 20 which represent assay conditions feasible on a benchtop NMR instrument when the K_M of the substrate is in the few tenth μM range were simulated. The concentration of the tested molecules is 200 μM which mimics the experimental conditions for fragment screening with the n-FABS. The horizontal dashed line in the three graphs is drawn at F = 15% and represents the detection limit. Only molecules displaying a F value larger than 15% are considered hits. The K_I detection limits are for the substrate competitive inhibitors 566.7, 103.0 and 54.0 μM for the ratios $[S]/K_M = 1$, 10 and 20, respectively, for the substrate uncompetitive inhibitors 566.7, 1030.3 and 1079.4 μM for the ratios $[S]/K_M = 1$, 10 and 20, respectively and for the noncompetitive inhibitors 1133.3 μM.

Signal enhancement by heteronuclear $^1H \rightarrow {}^{19}F$ NOE.

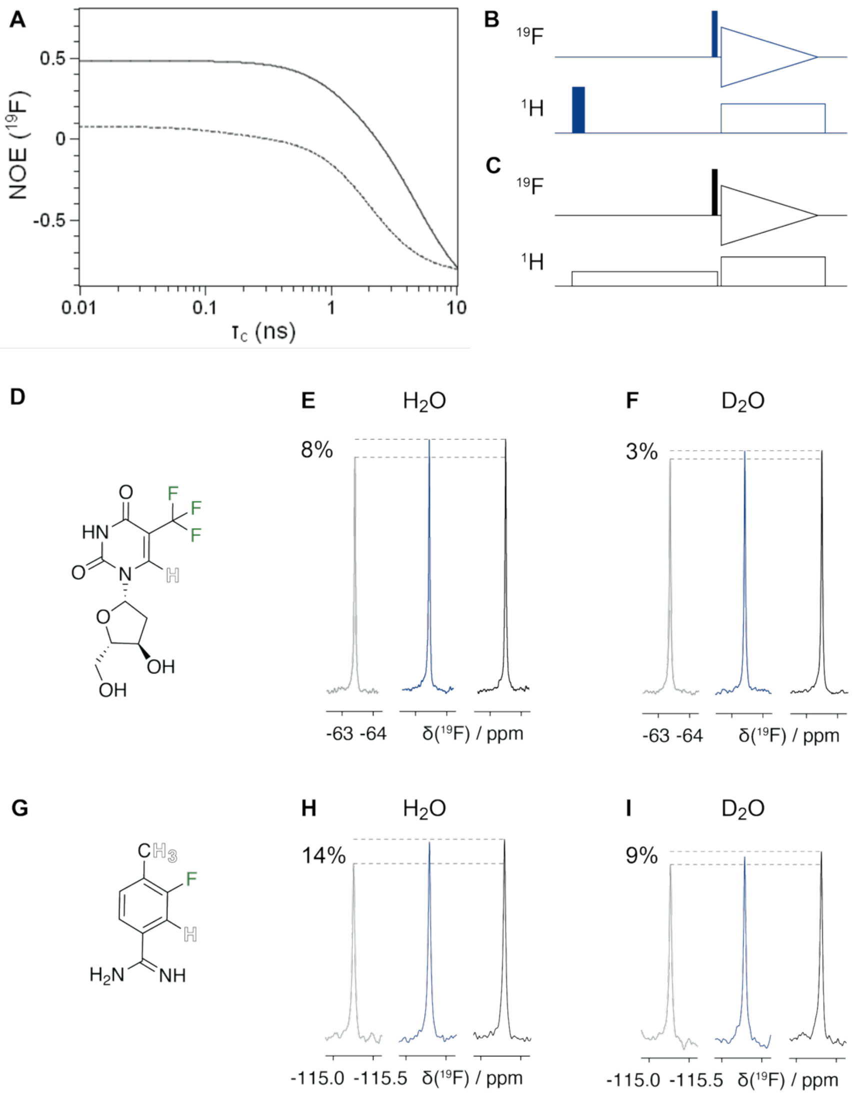

Suppl. Figure 2: Signal enhancement by heteronuclear $^1H \rightarrow {}^{19}F$ NOE. (A) Plot of the theoretically achievable heteronuclear $^1H \rightarrow {}^{19}F$ NOE as a function of the correlation time τ_c derived from Eq (6) in Dalvit & Piotto, MRC 2016, 55, 106–114. The simulations were performed for a 600 MHz (dashed line), and for an 80 MHz (continuous line) NMR spectrometer for an H-F spin system with $r_{FH} = 2.6$ Å, $\Delta\sigma = 76.8$ ppm and $\eta_{CSA} = 0$. The x axis is on a logarithmic scale. Pulse sequences used for $^1H \rightarrow {}^{19}F$ NOE via inversion of ^1H followed by a delay τ before the detection (B) or saturation of ^1H applied for a defined delay before the detection (C). Narrow and wide filled rectangles represent 90° and 180° pulses, respectively, and empty rectangles represent pulse trains

for decoupling and saturation (Waltz16). NMR spectra were recorded in 90% H_2O/10% D_2O (E, H) and pure D_2O (F, I) using a pulse-acquire experiment as reference (pulse sequence not shown, grey signal), pulse sequence B (blue) and pulse sequence C (black). Spectra were recorded at 298 K on the 80 MHz instrument (Bruker Fourier80). The recycle delay and the delay τ were set to 5 s and 1 s, respectively. The concentration of the molecules was 10 mM and 256 scans were recorded. The structure of TFT is shown in D, with hydrogen atoms in proximity of the detected fluorine atoms outlined. Since the ^{1}H to ^{19}F ratio in TFT is unfavourable, an alternative molecule was tested (G). The resulting enhancements due to NOE are indicated next to the spectra.

Example of IC_{50} fitting procedure with equation 8 on TP0006

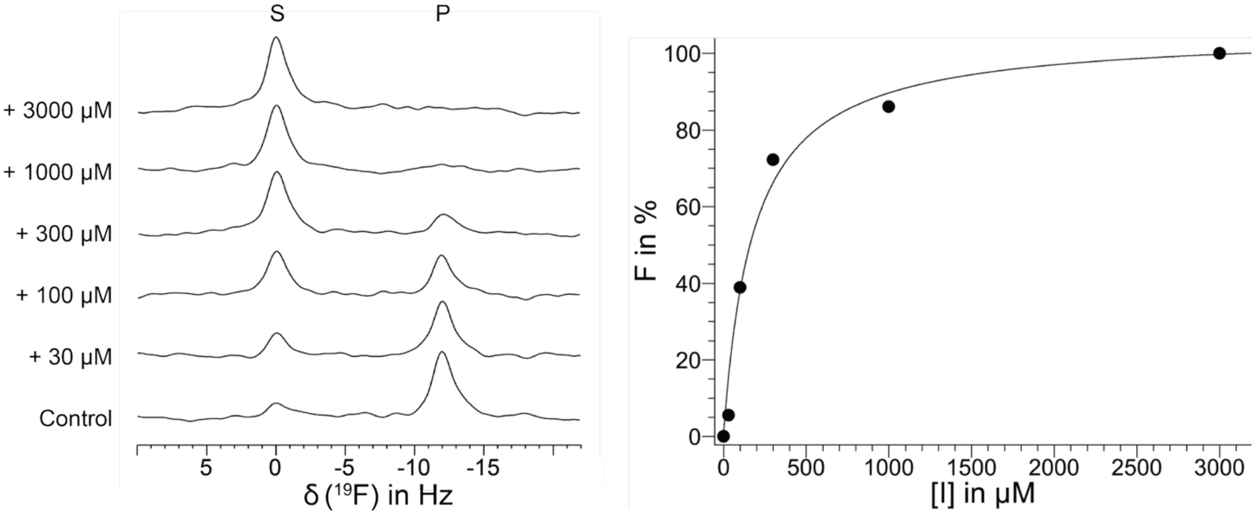

Suppl. Figure 3: Example of fitting procedure with equations 8 on TP0006. On the left, the spectra used for extracting substrate signal intensity are shown. The diagrams on the right show fits of the experimental data with equation 8.

IC_{50} values determined with different fitting parameters

Suppl. Table 1: Results from fitting IC_{50} values while keeping the parameters n and P fixed or allowed to float. The data and inhibitor concentrations shown in Figure 5 were used to fit IC_{50} values. The IC_{50} data reported in Figure 5 are the ones from the first column, where the variables n = 1 and P = 100 were fixed. For validation of the fitting procedure, n was allowed to float (column 2) and n and P were both allowed to float (last column).

**Molecule**	**Eq. 8 (n = 1, P = 100)**	**Eq. 8 (n = 1)**	**Eq. 8**
TP0001	IC_{50} = 0.029 ± 0.007 μM	IC_{50} = 0.042 uM*<br>P = 113.9	IC_{50}= 0.046 uM*<br>n=0.91<br>P=118.0
TP0004	IC_{50} = 72.2 ± 4.1 μM	IC_{50} = 78.6 ± 4.0 μM<br>P = 102.9 ± 1.1	IC_{50} = 75.5 ± 2.2 μM<br>n = 1.11 ± 0.04<br>P = 100.9 ± 0.8
TP0005	IC_{50} = 7.0 ± 0.7 μM	IC_{50} = 6.4 ± 0.9 μM<br>P = 97.3 ± 3.0	IC_{50} = 6.6 ± 1.3 μM<br>n = 0.93 ± 0.19<br>P = 98.7 ± 5.5
TP0006	IC_{50} = 154 ± 25 μM	IC_{50} = 178.6 ± 39.4 μM<br>P = 105.6 ± 6.0	IC_{50} = 142 ± 21 μM<br>n = 1.42 ± 0.25<br>P = 97.0 ± 4.7

* For the compound TP0001 no meaningful error estimate could be determined, since it is at the lower limit of the assay window.

Pulse sequence

```
;FAXS3sp_1H19FNOE_Neo.mp
;avance-version (01/02/2025)
;1D FAXS with 19F spin echo
;with 1H/19F Heteronuclear NOE module using 1H inversion
;Using two distinct amplifiers for 1H and 19F
;No adiabatic pulses

;$CLASS=HighRes
;$DIM=1D
;$TYPE=
;$SUBTYPE=
;$COMMENT=


;$OWNER=mp
#include <Avance.incl>
#include <Grad.incl>
#include <Delay.incl>


"d11=30m"
"p2=p1*2"
"d12=20u"

"DELTA=d1-100m"
"acqt0=0"

1 ze
  d11 pl1:f1
  d11 pl12:f2
2 30m do:f2
  d12 UNBLKGRAMP
  d12 LOCKH_OFF
  DELTA
  10u pl12:f2
  100m
  d12 BLKGRAMP
  d12 LOCKH_ON
  d12 pl1:f1 pl2:f2
  (p3*2 ph4):f2
  4u
  p16:gp1
  d16 pl12:f2
  1m
  10u gron0
  d30
  20u groff
  4u
  p1 ph1
3 d20
  (p2 ph2):f1
  d20
  lo to 3 times l3
  go=2 ph31 cpd2:f2
  30m do:f2 mc #0 to 2 F0(zd)
  d12 UNBLKGRAMP
  d12 LOCKH_OFF
exit


ph1=0 0 2 2 1 1 3 3
ph2=1 3 1 3 0 2 0 2
ph3=0
ph4=0
ph31=0 0 2 2 1 1 3 3
```

```
;pl1 : f1 channel - power level for pulse (default)
;pl12: f2 channel - power level for CPD/BB decoupling
;pl13: f2 channel - power level for second CPD/BB decoupling
;p1 : f1 channel -  high power pulse
;d1 : relaxation delay; 1-5 * T1
;d11: delay for disk I/O                             [30 msec]
;d20: fixed echo time
;NS: 1 * n, total number of scans: NS * TD0
;cpd2: decoupling according to sequence defined by cpdprg2
;pcpd2: f2 channel - 90 degree pulse for decoupling sequence

;use gradient ratio:   gp 1 : gp 2

;for z-only gradients:
;gpz0: 0.2%
;gpz1: 30%


;use gradient files:
;gpnam1: SMSQ10.100
```
